# Mechanisms and Efficacy of Intravenous Lipid Emulsion Treatment for Systemic Toxicity From Local Anesthetics

**DOI:** 10.3389/fmed.2021.756866

**Published:** 2021-11-08

**Authors:** Yang Liu, Jing Zhang, Peng Yu, Jiangfeng Niu, Shuchun Yu

**Affiliations:** ^1^Department of Anesthesiology, The Second Affiliated Hospital of Nanchang University, Nanchang, China; ^2^Key Laboratory of Anesthesiology of Jiangxi Province, Nanchang, China; ^3^Department of Endocrinology and Metabolism, The Second Affiliated Hospital of Nanchang University, Nanchang, China

**Keywords:** local anesthetic systemic toxicity, lipid emulsion, mechanism, resuscitation, bupivacaine

## Abstract

Local anesthetics are widely used clinically for perioperative analgesia to achieve comfort in medical treatment. However, when the concentration of local anesthetics in the blood exceeds the tolerance of the body, local anesthetic systemic toxicity (LAST) will occur. With the development and popularization of positioning technology under direct ultrasound, the risks and cases of LAST associated with direct entry of the anesthetic into the blood vessel have been reduced. Clinical occurrence of LAST usually presents as a series of severe toxic reactions such as myocardial depression, which is life-threatening. In addition to basic life support (airway management, advanced cardiac life support, etc.), intravenous lipid emulsion (ILE) has been introduced as a treatment option in recent years and has gradually become the first-line treatment for LAST. This review introduces the mechanisms of LAST and identifies the clinical symptoms displayed by the central nervous system and cardiovascular system. The paper features the multimodal mechanism of LAST reversal by ILE, describes research progress in the field, and identifies other anesthetics involved in the resuscitation process of LAST. Finally, the review presents key issues in lipid therapy. Although ILE has achieved notable success in the treatment of LAST, adverse reactions and contraindications also exist; therefore, ILE requires a high degree of attention during use. More in-depth research on the treatment mechanism of ILE, the resuscitation dosage and method of ILE, and the combined use with other resuscitation measures is needed to improve the efficacy and safety of clinical resuscitation after LAST in the future.

## Introduction

In the 1880s, cocaine was first used as a local anesthetic in ophthalmology and dental anesthesia (1). Since then, local anesthetics have become widely used clinically and are considered indispensable. However, local anesthetic poisoning incidents caused by the mistaken entry of local anesthetics into blood vessels can occur. Fortunately, the use of ultrasound technology has greatly reduced, but not eliminated, the occurrence and the risk of such incidents. In addition to a series of preventive measures such as using the lowest dose of local anesthetics and precise injection of anesthesiology drugs according to the basic situation of the patient, the timely detection and the treatment of the systemic toxicity from local anesthetics are crucial. The deadliest symptoms of local anesthetic systemic toxicity (LAST) are suffocation caused by airway spasm and heart failure caused by myocardial suppression. Therefore, reasonable early approaches to LAST treatment involve operations for airway management and advanced life support (2). In 1997, Weinberg et al. (3) pretreated rats with bupivacaine-induced cardiac arrest with a lipid infusion and found that the time to cardiac arrest was prolonged and the recovery time was earlier. As a result, lipid emulsion has become known as not only a parenteral nutrition option but also a treatment for LAST; with the publication of many experimental results, it has now become the first-line treatment of LAST. However, its mechanism of action is still unclear.

## Local Anesthetic Systemic Toxicity (LAST)

### Local Anesthetics

Local anesthesia temporarily blocks the conducting function of the nerve plexus so that the corresponding area innervated by the nerve plexus loses sensation. Local anesthesia is simple and easy to perform. It is often used clinically, alone or in combination with general anesthesia, to reduce the use of opioids after surgery and to provide patients with satisfactory perioperative analgesia. When the concentration of local anesthetics in the blood exceeds the tolerance of the body, although, corresponding systemic toxicity that excites or inhibits the central nervous system (CNS) and cardiovascular system will occur. If the dose is too large, malignant arrhythmia and severe myocardial depression may occur, and cardiovascular failure will eventually endanger life and safety (4). Ultrasound-directed positioning technology has greatly improved the safety and accuracy of local anesthesia. Ultrasound reduces the incidence of vascular puncture related to PNB (peripheral nerve block) and improves the efficacy of PNB. Barrington et al. (5) and Abrahams et al. (6) found through statistical analysis that the use of ultrasound guidance was related to a decrease in the incidence of local anesthetic toxicity (from mildly toxic subjective symptoms to epileptic seizures and cardiac arrest). The type and the dosage of the local anesthetic, the proficiency of the nerve block technique, the patient's own physical fitness, the time when a LAST event is discovered, the appropriateness of treatment measures, and other considerations are all related to the success of the local anesthesia and, more importantly, to the question of whether the safety of the patients is guaranteed (7, 8).

### Toxicity Mechanisms

Local anesthetics work by combining with voltage-gated Na^+^ channels to block their opening and inhibit the inflow of Na^+^, thereby hindering the depolarization of action potentials to exert anesthetic effects. When the concentration of local anesthetics is too high, local anesthetics will act on Na^+^, K^+^, Ca^2+^, Na^+^-K^+^-ATPase channels, and other targets to interfere with the signal transduction of intracellular and transmembrane cells. This interference thereby inhibits the metabolism of cyclic adenosine monophosphate (cAMP), protein kinase B, adenylate-dependent protein kinase (AMPK), and other molecules related to cell survival and apoptosis (9).

### CNS Toxicity

The central nervous system is the system most sensitive to high concentrations of local anesthetics in the blood. The threshold plasma concentration of local anesthetics used to induce CNS toxicity is lower than that used to trigger cardiovascular toxicity, so CNS symptoms appear earlier than those in the cardiovascular system (10, 11).

Under normal circumstances, the CNS inhibition and excitation systems of the body interact to maintain balance. However, when the concentration of the local anesthetic in the plasma is excessive, the anesthetic quickly enters the blood-brain barrier and destroys the inhibitory neurons of the cerebral cortex, in part by blocking Na^+^ channels. This blockade causes obstacles to depolarization. First, excitatory CNS poisoning occurs. The clinical manifestations begin with abnormalities in the cranial nerves, such as changes to sight, hearing, and smell. Dizziness, tinnitus, mouth and tongue numbness, and a metallic taste can develop (12), and manifestations can progress into epilepsy symptoms of persistent muscle contraction and abnormal discharge of brain neurons (13). Increased concentrations of local anesthetics in the plasma inhibit the CNS, and the patient experiences a change in symptoms from those related to the excited phase to those of the inhibited phase: the consciousness gradually disappears, and the patient falls into a deep coma and finally stops breathing (9).

### Cardiovascular System Toxicity

Toxicity to the cardiovascular system appears later; however, after symptoms appear, the overall health of the patient is more seriously impaired. Cardiotoxicity from local anesthetic poisoning mainly results from negative effects of the anesthetic on myocardial contractility, cardiac conduction, and systemic vascular resistance. Local anesthetics block the Ca^2+^ channel and Na^+^-Ca^2+^ exchange pump of cardiomyocytes, reducing the intracellular calcium storage; thus, the force of the myocardial contraction decreases. At the same time, as transmembrane cell transduction pathways are interrupted, the levels of PKB and AMPK are reduced, which reduce the storage of adenosine triphosphate (ATP) and cAMP in the cell. As a result, the metabolic level of myocardial cells is reduced, and the contractility of the myocardium is reduced even more. The conduction of the myocardium is also blocked; the Na^+^ channel is blocked; and the absolute value of the intracellular resting potential increases, which leads to the prolongation of the PR, QRS, and ST intervals. As the concentration of local anesthetics in the plasma increases even more, the K^+^ channel becomes blocked, the QT interval is prolonged, and rapid and slow arrhythmias occur (9). Eventually, the heart is arrested.

Among the local anesthetics, bupivacaine is associated with a great number of cardiac arrests; generally, the more fat-soluble local anesthetics are more likely to cause cardiovascular failure. In dental uses, lidocaine accounts for the majority of LAST experiences, perhaps because lidocaine is used very frequently in dental procedures. It is unlikely that lidocaine is more toxic than bupivacaine (14). However, the use of bupivacaine in obstetrics is higher, and bupivacaine crosses placental barrier and so threatens the safety of the mother and the baby. Therefore, we should be familiar with the toxic doses of various local anesthetics and use the lowest effective dose to prevent LAST.

The number of recorded LAST incidents is decreasing, and most of them occur in smaller hospital settings, such as clinics and outpatient surgery centers. Because non-anesthesiologists use local anesthetics after operations, and because non-anesthesiologists make judgments about LAST, a certain degree of misjudgment and missed judgments likely exists about the reduction of LAST events. Whether doctors have complete judgments about the individualized use of local anesthetics and first aid remains unclear (12). Anesthesiologists must be familiar with the peripheral nerve anatomy and the pharmacological properties of local anesthetics, and they must correctly implement the local anesthetic technique. All professional medical personnel who are qualified to use local anesthetics must be strictly trained to reduce the occurrence of LAST (15).

Clinical cases and experimental studies have proved that ILE reverses systemic toxicity caused by local anesthetic overdose (16–19). A recent meta-analysis has also supported the use of ILE to improve survival in animal models of bupivacaine poisoning (20). For all patients with local anesthetic overdose, ILE can improve the symptoms to a certain extent (21). The American Society of Regional Anesthesia and Pain Medicine (ASRA), updated in 2017, emphasized the importance of lipid emulsions in the LAST management checklist, indicating that prioritizing airway management and early infusion of lipid emulsions can effectively prevent the vicious circle of hypoxia and acidosis symptoms in the late stage of poisoning (22). Therefore, hospitals that use local anesthetics for regional anesthesia or pain management should stock lipid emulsion preparations (23, 24).

## Lipid Emulsion Therapy in LAST

### Lipid Emulsions

Clinically, lipid emulsions have gradually become an economical and easy-to-obtain preparation. Since the 1960s, lipid emulsions have been used in parenteral nutrition preparations. Recently, it has appeared in emergency departments and intensive care units. Lipid emulsion has a significant rescue effect on patients with acute fat-soluble drug poisoning, a benefit that has been identified by many clinical guidelines (25–27). Lipid emulsions can rescue patients with acute poisoning of fatsoluble drugs, including antiarrhythmic drugs, psychotropic drugs, antimalarial drugs, and organophosphates (28–31). In Rosenblatt et al. (32) used 20% lipid emulsion infusion and successfully resuscitated a patient whose heart was arrested for a long time as a result of bupivacaine toxicity. Thus, the effectiveness of lipid emulsions has been verified in clinical cases; many reports have documented successful recovery from LAST with lipid emulsions (33–35). Lipid emulsion therapy has also achieved good results as a treatment for LAST in newborns and children, not just in adults (36, 37). Fast-track lipid rescue is strongly recommended when serious complications like cardiac arrest and heart failure occur, especially in infants who are sensitive to ischemia and hypoxia of the cardiovascular system. According to the 2015 Special Situation Resuscitation Guidelines from the American Heart Association, lipid emulsion therapy is recommended as an adjuvant therapy to highly effective cardiac life support measures for cardiac arrest induced by LAST (38). At present, ILE has also been widely used in the resuscitation process of nerve and cardiotoxicity caused by overdose of local anesthetics.

### Other Anesthetics Participate in the Resuscitation Process of LAST

Nonselective opioid receptor antagonists, such as naloxone, are used to treat acute intoxication from narcotic analgesics, or for detoxification of central depression caused by opioids after surgery. Selective δ and κ opioid receptor antagonists can antagonize the resuscitation of lipid emulsion on cardiotoxicity, whereas selective μ opioid receptor antagonists will not disrupt the effects of lipid emulsion (39).

Dexmedetomidine is a highly selective α2 adrenergic receptor agonist used for intraoperative sedation and auxiliary analgesia. It can reduce the spinal cord neurotoxicity induced by lidocaine by inhibiting glutamate release and protein kinase C pathways (40); these mechanisms are similar to those that drive the rescue of neurotoxicity by lipid emulsion. At the same time, dexmedetomidine reduces the cardiotoxicity caused by bupivacaine, but α2 receptors are not involved in this process (41). The mechanism of action for dexmedetomidine is still unclear and needs additional investigation.

Propofol can be used as an anticonvulsant sedative treatment. Propofol reduces the level of excitatory amino acids, reduces the activity of nitric oxide synthase, blocks the synthesis of nitric oxide, reduces the cerebral cortex spike reflex, and reduces the brain wave activity that controls the occurrence of epilepsy by inhibiting convulsions (42). Inhibition of nitric oxide release alleviates the toxicity to the CNS and cardiovascular system caused by an overdose of local anesthetics. Injection propofol (as a solution containing 10% soybean oil, 1.2% purified lecithin, and 2.25% glycerin) before ILE administration to patients with LAST is not effective to reverse the LAST. In experiments on isolated rat hearts, low lipid concentrations did not significantly reduce the bupivacaine level and did not reverse the bupivacaine-induced reduction of heart function (43). Propofol has a significant inhibitory effect on the respiratory system and the cardiovascular system, so propofol cannot be used to replace lipid emulsion treatment; Propofol is likely to inhibit cardiovascular function even more, which is counterproductive (15).

Although the mechanism of dexmedetomidine and propofol in neurotoxicity is not clear, studies have shown that the use of lipid emulsion combined with other anesthetics can improve the resuscitation effect. Opioid receptor antagonists will antagonize this resuscitation effect (44).

### Research Progress on the Use of Lipid Emulsion

Recent studies on the infusion program of lipid emulsion to resuscitation of bupivacaine-induced cardiac arrest in rats have shown that split intravenous infusion has a better resuscitation effect than continuous intravenous infusion (45). In addition, the composition of the lipid emulsion will have different effects on the cardiovascular system. Experiments have proved that long-chain triglyceride preparations, such as lipid emulsion, are more effective in metabolism than mixed long-chain/medium-chain triglyceride (LCT/MCT) preparations, and the pharmacokinetic properties of lipid emulsions are better than those of LCT/MCT emulsions (46). Kim et al. (47) proved that LCT/MCT emulsion was stronger than LCT emulsion alone as a treatment for acute bupivacaine poisoning. MCT enhanced the metabolism of local anesthetics, and LCT has a stronger ability to combine local anesthetics. LCT/MCT emulsion improved cardiac systolic function better than LCT emulsion alone. This analysis showed that the ratio of MCT and LCT in lipid emulsion directly affects the resuscitation effect, and future studies must clarify the appropriate ratio.

## Multimodal Mechanism of LAST Reversal by Lipid Emulsion

### Lipid Sink

Lipid emulsion enters the blood system to form a lipid depot, which can absorb highly fat-soluble drugs (local anesthetics and nonlocal anesthetics) to clear them from the tissues and organs where the drugs are deposited, thereby improving cardiovascular function. A widely accepted mechanism for this absorption is the lipid sink theory (48). As a highly fat-soluble local anesthetic, bupivacaine combines with lipid molecules, and this binding accelerates the clearance of bupivacaine in the isolated rat heart and allows the heart to recover its spontaneous contractions faster than without binding (49). Experiments support the lipid sink theory, but these data results cannot rule out the existence of other mechanisms of action. In addition, lipid emulsion pretreatment can reduce the cardiotoxicity caused by bupivacaine, prolong the occurrence time of cardiac arrest, and shorten the time of cardiac recovery. However, pretreatment mepivacaine at the same dose did not significantly change the time of recovery from cardiac arrest, which indicates that the pharmacokinetic properties of local anesthetics, such as fat solubility, may affect the success of lipid emulsion pretreatment (50). In addition to lipophilicity, electrostatic attraction may play a role in treatment success. For example, a fat-soluble local anesthetic is electrolytically positive, and it binds to negatively charged lipid particles. This hypothesis is also an auxiliary view of the following lipid shuttle theory (12).

### Lipid Shuttle

Lipid shuttle refers to the way that the lipid emulsion, like a subway shuttle, moves back and forth; combined with local anesthetics, this movement accelerates the uptake of the liver of the anesthetic and promotes the redistribution of local anesthetics, thereby enhancing the metabolism of the anesthetic and alleviating cardiotoxicity. The heart and the brain are highly sensitive to decreases in blood volume and oxygen content. The lipid emulsion removes the local anesthetic from the heart and brain and redistributes it to the liver and muscle tissues for acute detoxification and digestion (51). The total plasma half-life of bupivacaine mainly depends on the time it takes to reach equilibrium in the tissues, because the metabolic process of bupivacaine is much slower than its distribution in the tissues. In research by Litonius et al. (52), lipid emulsion reduced the total plasma half-life of bupivacaine, suggesting that bupivacaine distributed to the tissues quickly. Another experiment focused on analyzing the effect of lipid emulsion administration on the pharmacokinetics and tissue distribution of bupivacaine. The content of bupivacaine in the heart, brain, lung, kidney, and spleen of the lipid emulsion group was lower than that in the control group. The content of bupivacaine in the liver was higher than that in the control group at 20, 30, and 45 min (53). These results showed that lipid emulsion promoted the transport and uptake of bupivacaine to the liver. Liver metabolism of bupivacaine may decrease its plasma concentration. Lipid shuttle is one of the mechanisms of lipid emulsion recovery, and this effect may not be limited to bupivacaine.

**Figure 1:**
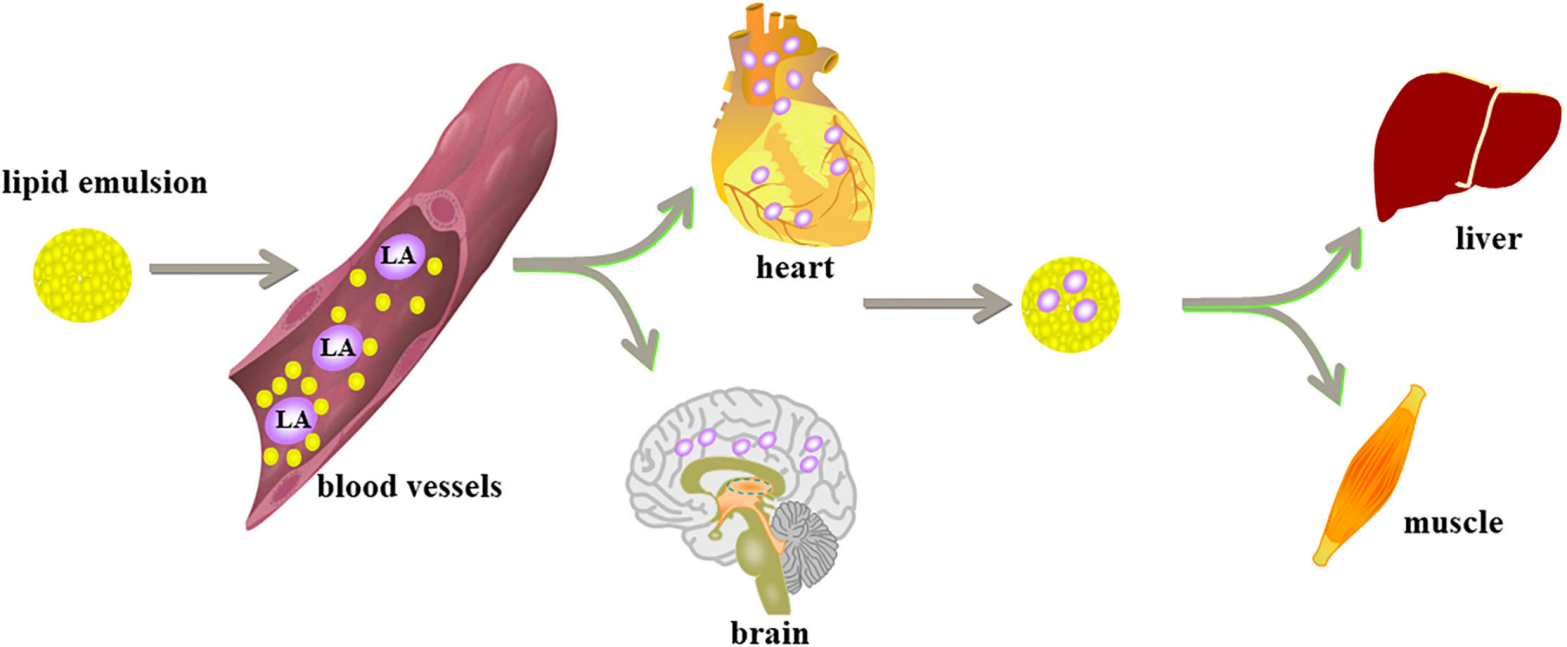
Lipid emulsion enters the blood system to form “lipid sink” and wrap local anesthetics and remove them from various tissues and organs. Lipid emulsion is like a shuttle subway. It combines local anesthetics in the heart and the brain, which are most sensitive to the decline of blood oxygen, and is distributed to the liver and muscle tissue to begin detoxification and digestion.

Free local anesthetic exerts the toxic effects on the CNS and cardiovascular system. However, according to the “lipid sink” theory, lipid emulsion will collect the local anesthetics deposited in tissues and organs into the blood vessels. At this time, the concentration of free local anesthetics in the blood vessels increases. Does this mean that lipid emulsion will increase the systemic toxicity of local anesthetics? Of course not—this is just a brief ascent process. A study has shown that the infusion of lipid emulsion can reduce the peak concentration of local anesthetics by 26–30% (54); these findings prove that the lipid emulsion only temporarily absorbs the local anesthetic from the heart and brain organs into the blood, which temporarily increases the concentration of local anesthetics in the intravascular space. Then, lipid emulsion transports the local anesthetics to the liver to accelerate their clearance. This temporary increase in local anesthetics intravascularly does not aggravate the systemic toxicity of local anesthetics (55). “Lipid sink” and “lipid shuttle” are the two main theories for lipid emulsion therapy. Animal experiments and clinical cases support them (Table 1). Their mechanisms have been accepted by people (Figure 1).

**Table 1:** The human clinical trials and animal experiments support the “lipid sink” and “lipid shuttle” theory.

**References**	**Models**	**Local anesthetics**	**Lipid emulsion**	**Observation index**	**Effect**
Litonius et al. (52)	8 males	Bupivacaine (0.5 mg/kg) over 20 min	intralipid^®^ for 30 min	context-sensitive half-life of total plasma bupivacaine from 45 (95% CI 32–76) min to 25 (95% CI 20–33) min	Decreased. The distribution of bupivacaine in the tissues slightly increased.
Heinonen et al. (56)	8 males	Lidocaine 1.0 mg/kg	1.5 ml/ kg bolus of 20% intralipid^®^ (200 mg/ml)	AUC_0−30*min*_ of lidocaine	Decreased. The distribution of lidocaine in the tissues increased and the volume of distribution increased.
Dureau et al. (57)	8 males and 8 females	ropivacaine or levobupivacaine (8 mg/min up to 120 mg)	120 ml 20% intralipid^®^	blood ropivacaine and levobupivacaine concentrations	Peak concentration was decreased by 26 to 30%.
Chen et al. (58)	12 patients undergoing below-knee surgery for a fracture	0.375% levobupivacaine (2.5 mg/kg)	pretreatment with 1.5 ml/kg intralipid^®^	total and free maximum plasma levobupivacaine concentrations	The apparent volume of distribution and clearance levobupivacaine increased, and the maximum total free concentration decreased.
Weinburg et al. (3)	rats	0.75% bupivacaine hydrochloride at a rate of 10 ml × kg × min^−1^	10, 20, or 30% Intralipid	Median doses of bupivacaine (in milligrams per kilogram)	Lipid infusion shifts the dose-response to bupivacaine-induced asystole.
Weinburg et al. (49)	rats	500 micromol/L bupivacaine	1% lipid emulsion	Average (±SEM) times to first beat and to 90% recovery of rate pressure product	Lipid emulsion speeds loss of bupivacaine from cardiac tissue while accelerating recovery from bupivacaine-induced asystole.
Chen et al. (59)	rats	40 μM bupivacaine	a range of lipid concentrations (0–16%)	The ratio of the maximum rate pressure product during recovery to baseline value	Lipid application in bupivacaine-induced asystole displays a concentration-dependent and time-response relationship.
Aumeier et al. (50)	rats	bupivacaine (250 μM) or mepivacaine (1,000 μM)	lipid emulsion (0.25 ml kg^−1^ min^−1^	times from start of perfusion LA to a 1 min period of asystole and recovery	Pretreatment with lipid emulsion extended the time until occurrence of asystole and decreased times to recovery in bupivacaine-induced cardiac toxicity but not in mepivacaine-induced cardiac toxicity.

### Inhibition of the Opening of the Mitochondrial Membrane Permeability Transition Pore

Myocardial cells contain a variety of sulfur kinases, which can catalyze the conversion of fatty acids with different lengths of carbon chains into acyl coenzyme A. Thus, myocardial cells preferentially use the oxidative decomposition function of fatty acids. Bupivacaine inhibits the action of carnitine-acylcarnitine translocase on the inner mitochondrial membrane, thereby inhibiting fatty acid beta oxidation. In the process of oxidation, ATP production is greatly reduced; acetyl coenzyme A is also reduced, and the oxidation of pyruvate in mitochondria of myocardial cells is also inhibited (60). Therefore, bupivacaine may inhibit the metabolism of fatty acids by inhibiting mitochondrial function. The function of mitochondria is closely related to energy metabolism. Lipid emulsion has rescued rats with bupivacaine poisoning when they were pretreated with a low-dose fatty acid oxidation inhibitor (61). The treatment improved the heart rate, the cardiac ejection fraction, and other cardiac function indexes, and this rescue mechanism was related to the inhibition of the membrane permeability transition pore (MPTP) opening. After bupivacaine treatment, the release of cytochrome C from the mitochondria to cytoplasm increased, and the ratio of Bax/Bcl-2 increased; these changes proved that the mitochondrial function of cardiomyocytes was impaired and that apoptosis increased; after lipid emulsion treatment, the release of cytochrome C decreased, and MPTP was inhibited (62).

The opening of MPTP is the key step of programmed cell death. Lipid emulsion can restore mitochondrial function by inhibiting the opening of MPTP, thus restoring the process of fatty acid metabolism. Rahman et al. (63) showed that lipid emulsion at the beginning of reperfusion could reduce the infarct size by approximately 70% and significantly improve the functional recovery of the heart. After ischemia, liposome could inhibit the opening of the MPTP, protect cardiomyocytes from bupivacaine-induced apoptosis, and protect against oxidative stress-induced cell injury. At the cellular level, the molecular mechanisms of local anesthetic poisoning and myocardial ischemia-reperfusion injury are similar (38).

### Activation of Ca^2+^ Channels to Enhance Myocardial Contractility

The intracellular Ca^2+^ homeostasis is jointly maintained by the action of mitochondria on the plasma membrane of the Ca^2+^ pump and the intracellular Ca^2+^ pool system. The pump transports Ca^2+^ from the cytoplasm to the mitochondria in an opposite concentration gradient, thereby maintaining the high Ca^2+^ balance in the mitochondria and the low Ca^2+^ balance in the cytoplasm. In an isolated rat heart model, the lipid emulsion increased the Ca^2+^ level in the myocardial cells, the left ventricular systolic pressure was significantly increased, and the myocardial contractility was enhanced (64). Chen et al. (65) showed that bupivacaine reduced the membrane potential of the mitochondria of cardiomyocytes and significantly reduced the free Ca^2+^ content in the mitochondria. The loss of Ca^2+^ caused mitochondrial dysfunction. The lipid emulsion can reverse this phenomenon, increase the stability of the membrane, regulate the concentration of calcium ions in the mitochondria, and maintain the production of ATP in the mitochondria. In addition, one of the important heart-strengthening effects of the lipid emulsion is provision of coronary blood perfusion. Only when the blood in the coronary artery brings the lipid emulsion to the myocardial area, where the local anesthetic concentration is high, can the local anesthetic level be reduced. Until the concentration of the myocardial local anesthetic decreases below the threshold value of Na^+^ and Ca^2+^ blocked by the local anesthetic, the toxicity cannot truly be reversed (12). After perfusion is established, increased cardiac output can enhance the efficiency of lipid emulsion in distributing local anesthetics to the liver and muscle tissue, forming a virtuous circle.

### Activation of the PI3K/AKt/GSK-3β Pathway

In the isolated rat heart model of myocardial ischemia-reperfusion, lipid emulsion significantly reduced the area of myocardial infarction and improved heart function (66). LY294002, a specific inhibitor of phosphoinositide 3-kinase (PI3K), completely blocked the protective effect of the lipid emulsion in the myocardium. PD98059, a specific inhibitor of extracellular signal-regulated kinase (ERK), can be partially blocked. Lipid emulsion can increase the phosphorylation levels of serine/threonine kinase (AKt), ERK, and glycogen synthase kinase-3β (GSK-3β). Therefore, lipid emulsion can reduce myocardial ischemia-reperfusion injury by activating the phosphorylation of downstream kinases (GSK-3β) mediated by upstream kinases (PI3K and AKt). This cell signaling pathway can also inhibit the MPTP, thereby reversing bupivacaine-induced cardiomyocyte apoptosis (63, 66). Michael et al. (67) also proved that glucose handling by Akt and AMPK is an indispensable part of the recovery from bupivacaine cardiotoxicity and the modulation of these pathways by ILE, which is conducive to lipid recovery.

### Inhibition of Nitric Oxide Release

**Figure 2:**
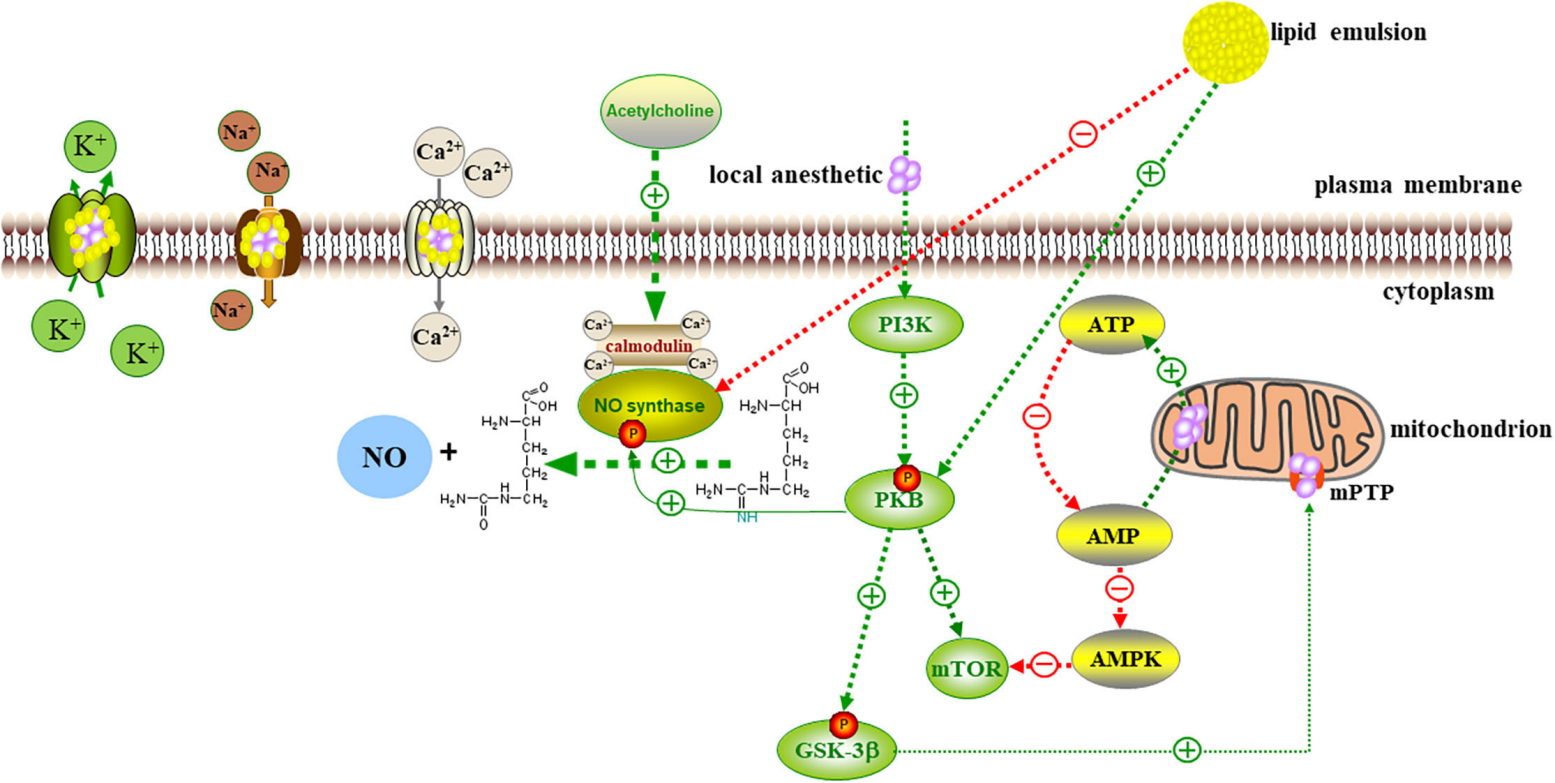
Local anesthetics act on Na^+^, K^+^, Ca^2+^ channels, interfere with signal transduction in cells and transmembrane cells, and inhibit the metabolic processes of PKB, AMPK, and other molecules related to cell survival and apoptosis. Local anesthetics increase the release of cytochrome c from mitochondria to cytoplasm, increased apoptosis, and impaired mitochondrial function. Lipid emulsion inhibited the opening of MPTP to restore mitochondrial function. Lipid emulsion can activate Ca^2+^ channels, increase the intracellular calcium level, and activate upstream kinase PI3K, and PKB-mediated downstream kinase GSK-3β Phosphorylation leads to the inhibition of MPTP opening, thereby reversing cardiomyocyte apoptosis induced by local anesthetics. Lipid emulsion inhibits the activation of eNOS, reduces the production and release of NO, and reverses the severe vasodilation caused by LAST.

The release of acetylcholine can stimulate the M3 choline receptor subtype of vascular endothelial cells, leading to the release of nitric oxide, the main active component of vascular endothelial relaxing factor, which causes the relaxation of vascular smooth muscle cells. In the aorta, with an intact endothelium, nitric oxide, prostacyclin, and more participate in the regulation of vascular tension through vasodilation; the contraction of the isolated vascular endothelium pretreated with epinephrine also depends on nitric oxide to weaken the contraction (68). Bupivacaine can inhibit protein kinase C and CPI-17 (a phosphorylation-dependent inhibitory protein of myosin), thereby inducing vasodilation in the isolated rat aorta, which is weakened after injection of lipid emulsion (69). Medium- and long-chain lipid emulsions can inhibit the activation of upstream endothelial nitric oxide synthase (eNOS) by guanylate cyclase in endothelial cells, thereby reducing the release of nitric oxide and inhibiting vasodilation (70). Lipid emulsion may reverse the severe vasodilation caused by LAST by inhibiting the activation of eNOS (71). In addition to the first two theories, the latter four mechanisms have gradually been recognized by experiments (Figure 2).

### Rebalance of the Excitation-Inhibition System

Central nervous system sensitivity to local anesthetics is much less than circulatory system sensitivity, but lipid infusion can also reverse the CNS symptoms of LAST. However, the metabolic hypothesis does not hold in the case of neurotoxicity (72–74). Xing et al. (75) proved that bupivacaine selectively damages the mitochondrial function of astrocytes, thereby inhibiting the uptake of glutamate and indirectly enhancing the calcium signal transduction in neurons induced by glutamate. Clinically relevant concentrations of bupivacaine increase the reactive oxygen species in astrocytes rather than neurons. The mitochondria are the core participants in the metabolic activities of astrocytes. In astrocytes, the dysfunction of mitochondrial respiratory chain, mitochondrial dynamics, and mitochondrial autophagy may lead to neurological diseases. Therefore, follow-up studies on the mechanism of CNS toxicity can focus on astrocytes. The mechanism of CNS toxicity from local anesthetics is fundamentally related to the imbalance of excitatory and inhibitory nervous system in the brain. Nie et al. (76) observed the CA1 pyramidal neuron current induced by glutamate and Gamma-aminobutyric acid (GABA) after lipid emulsion administered to treat CNS toxicity induced by bupivacaine and found that it can increase this current and release frequency. Lipid emulsion may balance the imbalance of the CNS by regulating the level of GABA; this hypothesis also provides a solution for the treatment of convulsions in the excitatory phase.

These mechanisms are related to each other, but they are relatively independent to explain the effects of lipid emulsion and cannot contain each other (24). Therefore, the mechanism of reversal of toxicity from local anesthetics by lipid emulsion is still considered multimodal, but the pharmacokinetic hypothesis of lipid emulsions has always occupied a major position in the assessment of possible mechanisms (77).

## The Issues of ILE Therapy in LAST

### Choice of Adrenaline in the Treatment of LAST

Adrenaline with a concentration of 1:200,000 is commonly used as an auxiliary drug in local anesthesia. Its vasoconstrictor effect can slow down the concentration of local anesthetics absorbed into the blood, prolong the effect of local anesthetics, and reduce systemic toxicity. In cardiac arrest caused by LAST, epinephrine improves coronary perfusion by constricting blood vessels and increasing systemic vascular resistance; thus, the lipid emulsion can reach the myocardial tissue to clear the local anesthetic. However, high-dose epinephrine (>10 μg/kg) and vasopressin can cause hyperlactic acidemia and severe acidosis in the treatment of LAST and can inhibit the resuscitation effect mediated by lipid emulsion. High-dose epinephrine, whether infused with a lipid emulsion or not, will increase the possibility of inducing arrhythmia and even offset any benefits of a lipid infusion (48). Weinberg et al. (78) and Di Gregorio et al. (79) have shown that the outcome of hemodynamic and metabolic indexes during lipid resuscitation is better than adrenaline and vasopressin. The combined application of lipid and epinephrine can restore heart function better than a lipid emulsion or epinephrine alone (80). However, the time and the effect of adrenaline combined with a lipid emulsion to treat LAST remain unclear. Adrenaline is related to the changes in pulmonary vascular pressure. Lipid emulsion administration is given priority before the use of epinephrine; this approach reduces the myocardial concentration of bupivacaine and reduces left ventricular pressure and ventricular diastolic dysfunction, which can reduce the lung damage from bupivacaine-induced cardiac depression injury, reduce the incidence of pulmonary hemorrhage after resuscitation, and improve the survival rate (81).

The role of epinephrine in the process of resuscitation of LAST with a lipid emulsion may be related to the dose, timing, and method of administration. ASRA recommends an additional injection of 1-mg adrenaline in other cardiac arrest situations. However, in the case of arrest caused by LAST, the adrenaline dose should not exceed 1 μg/kg. At the same time, the use of vasopressin, calcium channel blockers, beta blockers, and other local anesthetics should be avoided (22).

### Key Points of Airway Management, Advanced Cardiac Life Support and Lipid Emulsion

Every aspect of the process of resuscitation from LAST is very important. Insufficient airway support, poor quality of basic life support, and use of the wrong dose or formulation of lipids may lead to failure of resuscitation (73). Because hypoxia, hypercapnia, and acidosis aggravate the symptoms of LAST and directly affect the success of all subsequent treatments, scientific gas road management is needed to ensure an adequate oxygen supply for patients (82). The ASRA guidelines recommend, after airway management, the initiation of lipid emulsion treatment after the first symptoms of systemic toxicity of local anesthetics appear or in any LAST event that is judged to be potentially serious (83) and emphasize that, when managing LAST, adequate basic resuscitation measures (oxygenation and cardiopulmonary resuscitation) and specific detoxification treatments are required (48). The key to dealing with local anesthetic-induced cardiac arrest is to restore coronary perfusion as soon as possible to improve myocardial contractility and maintain cardiac output (84). If the concentration of the local anesthetic in the tissue is too high or if the lipid emulsion is not administered in time, the coronary arteries will be occluded, hindering the transport of the lipid emulsion to the capillary bed of the coronary arteries; then, the local anesthetic will fail to clear and will continue to accumulate in the heart and impair cardiac function. If the patient's own cardiopulmonary function is poor or other serious diseases inhibit the effectiveness of treatment, he or she should be considered for extracorporeal membrane oxygenation (7). A meta-analysis of animal data should be considered sufficient at this time to support the use of lipid emulsion (combined with other resuscitation measures) to treat LAST, especially when it results from bupivacaine. However, lipid emulsion resuscitation has failed in the presence of asphyxia arrest, lack of cardiopulmonary resuscitation, and treatment delay (85). When LAST occurs, airway management should be the first measure to ensure optimal oxygenation and ventilation. Because lipid infusion must be circulated to the coronary vascular bed, high-quality basic life support is a necessary factor in lipid resuscitation in low output conditions, and lipid emulsion should be implemented together with standard resuscitation programs. ILE cannot substitute for the standard resuscitation protocol (86).

### Predictions of Seizures

Seizures usually precede CNS and cardiovascular system depression (15). Therefore, medical workers need to be aware when epileptic seizures occur that patients may develop CNS depression or impending cardiovascular failure (87). ASRA recommends benzodiazepines as the first-line treatment for epileptic seizures induced by local anesthetic poisoning. A 1-mg/kg intravenous injection of propofol can also effectively prevent lactic acid-induced seizures and convulsive muscle movements. However, propofol has a significant inhibitory effect on the respiratory and cardiovascular systems. For patients with severely inhibited cardiovascular systems or with cardiovascular diseases, benzodiazepines are recommended. Early control of epileptic seizures and airway intervention for the treatment of hypoxemia and acidosis can prevent cardiac arrest and restore system function as soon as possible (25).

### What to Pay Attention to When Using Lipid Emulsion

According to current treatment guidelines, adverse reactions to lipid infusions in healthy volunteers are rare. Most of the adverse reactions occur when recommended dose is exceeded, and most complications are related to high-dose lipid emulsions. According to the 2020 version of the first aid list of ASRA for LAST, lipid emulsion can continue for at least 15 min when patients are hemodynamically stable, but the maximum dose of lipid should not exceed 12 ml/kg. When patients are considered stable, observation should continue. Observation should last 2 h after seizures or 4–6 h after cardiovascular instability, as appropriate after cardiac arrest (21, 88, 89). Clinically, ILE has caused fat overload syndrome in older. The reasons may be related to decline in body function and a decreased ability to clear fat (90, 91). In addition, the amount of lipid emulsion should be carefully considered in patients with lipid metabolism and storage disorders (38). Common adverse reactions after ILE include hypertriglyceridemia, fat embolism, hypersensitivity and allergic reactions, infection, local venous irritation, acute pancreatitis, and electrolyte imbalance. The Lipid Emulsion Working Group found that the adverse reactions associated with acute intravenous infusion of lipid emulsion included acute kidney injury, cardiac arrest, mismatched ventilation and perfusion, acute lung injury, venous thromboembolism, allergies and allergic reactions, fat embolism, fat overload syndrome, pancreas inflammation, blockage of the extracorporeal circulation circuit, and increased susceptibility to infection (92). For patients with severe egg allergy, ILE administration is contraindicated (93).

Marwick et al. (94) reported a case of a patient who was accidentally given bupivacaine intravascularly during a brachial plexus block, which resulted in cardiac arrest. The patient was successfully resuscitated with cardiopulmonary resuscitation, supplemented with 20% lipid emulsion. Forty min after the infusion of the lipid emulsion, cardiotoxicity reappeared. However, given the insufficient lipid emulsion, the patient was successfully resuscitated with amiodarone and inotropic drugs. This case indicates that LAST may recur after the initial lipid rescue. This situation indicates that, if necessary, the doctor should be prepared to restart lipid therapy. The patient needs to be continually monitored for several hours after the initial lipid resuscitation. A sufficient amount of lipid emulsion should be prepared in advance in case the symptoms recur and continued use of the lipid emulsion is needed. ILE can interfere with some laboratory measurements and affect therapeutic drug monitoring. Therefore, blood samples can be collected in advance before lipid emulsion treatment.

To determine when to use a lipid emulsion and how much to use, a risk assessment should be performed to weigh the pros and cons. Even if a lipid emulsion can be used, monitoring of the blood triglyceride content in real time is needed to make accurate clinical judgments at any time.

## Summary and Outlook

As a first-line preparation for the treatment of local anesthetic toxicity, lipid emulsion is economical, easy to obtain, and widely used. After use, the incidence of intubation is reduced and the time spent in the intensive care unit is shortened. Therefore, lipid infusion can save costs (73). However, its specific mechanism is still unclear. The mechanism of cardiotoxicity caused by local anesthetics is similar to that of cardiac ischemia reperfusion at the cellular level, which can be used as a reference to explain the rescue mechanism of lipid emulsion. In clinical work, we should try to prevent the occurrence of LAST. If it occurs, we must actively take basic resuscitation measures and routinely administer ILE. Multimodal rescue is essential. In fact, the CNS symptoms appear earlier than the cardiovascular system and can be an excellent reminder for judging the timing of LAST. In the early stage of poisoning, we should actively deal with CNS symptoms such as convulsions and epilepsy, as soon as possible to improve the resuscitation effect. According to the current research, the optimal dosage and method of lipid emulsion and the potential adverse reactions still need to be studied and verified. Additional research will help us to develop a more efficient method for removal of local anesthetics by considering molecular signaling pathways. Then, lipid emulsion can safely guide clinical resuscitation. The AAGBI (Associated of Anaesthetists of Great Britain and Ireland guidelines recommend that a 20% lipid emulsion should be “available immediately in all areas where potential toxic doses of local anesthetics are used.” Overall, a need remains to expand awareness in all medical workers about the importance of lipid emulsion in LAST and to lay the foundation for national promotion of lipid emulsion.

## Author Contributions

YL wrote the manuscript. SY guided the planning. JZ and PY wrote the review manuscript. JN critically amended the manuscript. All the authors read and approved the final manuscript.

## Funding

This work was supported by the grants from the National Natural Science Foundation of China (81760038, 81760048, and 81760050) and the Natural Science Foundation of Jiangxi Province (20171BAB205029).

## Conflict of Interest

The authors declare that the research was conducted in the absence of any commercial or financial relationships that could be construed as a potential conflict of interest.

## Publisher's Note

All claims expressed in this article are solely those of the authors and do not necessarily represent those of their affiliated organizations, or those of the publisher, the editors and the reviewers. Any product that may be evaluated in this article, or claim that may be made by its manufacturer, is not guaranteed or endorsed by the publisher.
